# Why revision of total hip arthroplasty fails: a retrospective consecutive cohort study of 963 patients

**DOI:** 10.1302/2633-1462.72.BJO-2025-0295.R1

**Published:** 2026-02-03

**Authors:** Valentin Cascales, Constant Foissey, Marcelle Mercier, Remy Coulomb, Writing Commitee, Michel-Henri Fessy, Sébastien Lustig, Pascal Kouyoumdjian

**Affiliations:** 1 Department of Orthopaedic and Traumatological Surgery, Spine Surgery Unit, University Hospital of Nîmes (CHU), Nîmes, France; 2 Orthopaedic Surgery Department, Croix Rousse Hospital, Hospices Civils de Lyon, Lyon Cedex, France; 3 Orthopaedic Surgery Department, Hospital Lyon Sud, Oullins-Pierre-Bénite, France; 4 University Mechanical and Civil Engineering Laboratory, Montpellier, France

**Keywords:** Revision total hip arthroplasty, Prosthetic joint infection, Dislocation, Dual mobility cup, Constrained liner, Periprosthetic fracture, Risk factors, Failure analysis, revision of total hip arthroplasty, cohort study, Other infections, periprosthetic fractures, revision surgery, periprosthetic joint infection (PJI), Recurrent dislocation, logistic regression, aseptic loosening, acetabular cups

## Abstract

**Aims:**

The aims of this study are to identify the causes and independent risk factors for failure following revision total hip arthroplasty (RTHA).

**Methods:**

We conducted a retrospective multicentre cohort study involving 963 patients who underwent RTHA between January 2016 and December 2021 across three French university hospitals, with a minimum follow-up of two years. Data collected included demographic details, revision rank (R1= first revision, *R*2= second revision, *R* ≥ 3=third or subsequent revision), surgical variables, complications, reoperations, re-revisions, and mortality. RTHA failure was defined as any reoperation or re-revision. Multivariate logistic regression was used to determine independent risk factors for failure.

**Results:**

The mean patient age was 72 years (14 to 104), with 55% of patients being female. The most common indications for RTHA were aseptic loosening (35.6%), periprosthetic fracture (32.0%), periprosthetic joint infection (PJI; 15.2%), and dislocation (5.7%). Failure occurred in 135 patients (14.0%), most frequently due to PJI (53%), of which 61% were new infections. Among patients revised for dislocation, recurrent instability accounted for 43.7% of failures. Most failures (70%) occurred within one year of revision. Independent risk factors included age ≥ 75 years (odds ratio (OR) 0.61), revision rank ≥ 3 (OR 1.96), PJI (OR 2.0), dislocation (OR 2.86), use of revision (OR 2.38), and constrained acetabular cups (OR 5.38).

**Conclusion:**

Aseptic loosening remains the leading indication for revision surgery, while PJI is the principal cause of failure following RTHA, both as a new infection and as iterative failure. Recurrent dislocation continues to pose a complex challenge despite modern implant strategies, underscoring the need for meticulous surgical planning in high-risk patients.

Cite this article: *Bone Jt Open* 2026;7(2):148–157.

## Introduction

Total hip arthroplasty (THA) is increasingly performed owing to the ageing population and the expansion of indications towards younger and more active patients.^1^ While the initial aim of THA was to relieve pain in elderly patients with advanced coxarthrosis, expectations have evolved towards restoration of full function, including return to work and sporting activities,^2,3^ without restrictions imposed by the prosthesis.^4^ This change raises concern regarding implant longevity and the inevitable increase in revision total hip arthroplasty (RTHA).

In France, between January 2012 and December 2018, the number of THA procedures increased by 10.5%, encompassing both primary and revision procedures. In 2018, there were 150,060 primary THAs and 19,457 revisions, and these numbers are projected to almost double by 2050, reaching nearly 300,000 primary THAs annually. A 43% rise in RTHA is thus anticipated, amounting to approximately 28,000 procedures each year.^5^

Large national joint registries provide reliable data on THA outcomes but do not capture all aspects of revision surgery because of smaller numbers, difficulty in linking revisions to their primary procedures,^6^ and variability in terminology such as ‘revision’, ‘reoperation’, or ‘re-intervention’.^7^ Previous multicentre series in France have shown loosening to be the predominant cause of revision, while periprosthetic fracture and infection are increasingly reported.^8^

Revision surgery is associated with higher complication rates than primary procedures,^9,10^ longer hospital stays, greater costs,^11^ and up to a five-fold increased risk of further revision.^12,13^

The aim of this study was therefore to describe the demographic characteristics of patients undergoing RTHA, to analyze the causes of failure, and to identify independent risk factors for iterative failure. We hypothesized that infection and instability would be the main contributors to failure following revision THA.

## Methods

### Study design and participants

We conducted a retrospective multicentre cohort study at three French university hospitals (University Hospital of Nîmes (CHU), Montpellier, France; Croix Rousse Hospital, Hospices Civils de Lyon, Lyon Cedex, France; and Hospital Lyon Sud, Oullins-Pierre-Bénite, France) performing both primary and revision hip arthroplasty. All patients undergoing RTHA between January 2016 and December 2021 were eligible for inclusion, irrespective of aetiology. All patients received written information and gave non-opposition for the use of anonymized data for research purposes. The study protocol was approved by the local Bioethics Committee and Institutional Review Board (24/05/02).

Exclusion criteria were defined to ensure a homogeneous RTHA population and included: conversion or revision of hemiarthroplasty, isolated fixation of periprosthetic fractures, arthroplasty following failed internal fixation of femoral neck fractures, isolated debridement without exchange of bearing surfaces, and procedures that did not involve revision of any THA component. Patients without a minimum follow-up of two years were excluded from the final analysis.

Revision was defined as the exchange of one or more components of a THA. Reoperation was defined as any surgical procedure related to the operated hip that did not involve component exchange (e.g. evacuation of haematoma, fixation of periprosthetic fracture, iliopsoas tenotomy, arthrolysis, or closed reduction of dislocation). Re-revision was defined as an iterative revision following the failure of a prior revision. Planned two-stage procedures for periprosthetic joint infection (PJI) were not considered failures, unless an unplanned reoperation occurred between stages or re-implantation was not possible.

PJI was defined according to the Musculoskeletal Infection Society (MSIS) criteria.^14^ Failures were categorized as intraoperative, early (< one year) or late (> one year). Fixation anomaly referred to any intraoperative evidence of defective or insufficient implant anchorage, while ‘noises’ described audible abnormal mechanical sounds (such as clicking or squeaking) reported by patients or identified clinically.

The revision rank was classified according to the number of previous revisions: first revision (R1), second revision (R2), or third and subsequent revisions (R ≥ 3). Failure of RTHA was defined as any reoperation or re-revision.

### Study population

A total of 963 patients underwent RTHA between January 2016 and December 2021 and were included in the analysis. An additional 74 patients were excluded because of follow-up < two years or incomplete datasets, representing 7.1% of the initial cohort. The mean age at revision was 72 years (SD 13.2; 14 to 104), with 55% of patients being female. The mean BMI was 26.2 kg/m² (SD 5.3). The mean follow-up was 4.4 years (SD 1.8; maximum 9.6 years), and the mean interval from index THA to revision was 11.1 years (SD 9.8). Revision rank was R1 in 69%, R2 in 20.3%, and R ≥ 3 in 10.7%.

### Outcomes

Data collection: Patients were identified using PMSI (Programme de Médicalisation des Systèmes d’Information) national hospitalization databases in each centre, by applying French CCAM procedure codes for RTHA (see Supplementary Table i).

A senior surgeon at each centre (VC, CF, MM) collected data using a standardized proforma, generating a shared database. Operative reports, radiographs, and medical records were reviewed. The dataset included demographic details, year of index THA implantation, revision rank (R1, R2, R ≥ 3), RTHA aetiology, type of revision (full component revision, partial component revision, or modular component exchange only), surgical approach, implant type, fixation method, bearing surface, grafting, complications, reoperations, re-revisions, and mortality.

The causes of RTHA were classified as: loosening (including osteolysis and wear), periprosthetic fracture, PJI (with ‘new PJI’ denoting infection occurring after revision for a non-infectious cause), dislocation, adverse reactions to metal debris (ARMD), implant fracture, implant malposition, leg length discrepancy (LLD), prosthetic or soft-tissue impingement, fixation anomaly, heterotopic ossification, and noises. Revision, reoperation, death, and complications were categorized as intraoperative, early (< one year) or late (> one year). To identify risk factors for RTHA failure, variables of interest were compared between patients with and without failure.

### Statistical analysis

Statistical analysis was performed using EasyMedStat v. 3.27 (EasyMedStat, France). Numerical variables were expressed as means (SDs), and discrete variables as absolute and relative frequencies (%). Patients were classified into two groups: those with re-revision and reoperation failure compared with those without. Basic demographic data were compared between groups. Risk factors for RTHA failure were sought through multivariate logistic regression. Data were checked for multicollinearity with the Belsey-Kuh-Welsch technique. Normality and heteroscedasticity of continuous data were evaluated using the Shapiro-Wilk and Leve tests, respectively. Continuous results were compared using unpaired *t*-test, Welch’s *t*-test, or Mann-Whitney U test depending on the data distribution. Discrete results were compared using the chi-squared test of Fisher’s exact test depending on the sample size. The α risk was set at 5%, and two-tailed tests were used. Statistical significance was set at p < 0.05.

## Results

### Indication for revision

**Fig. 1:**
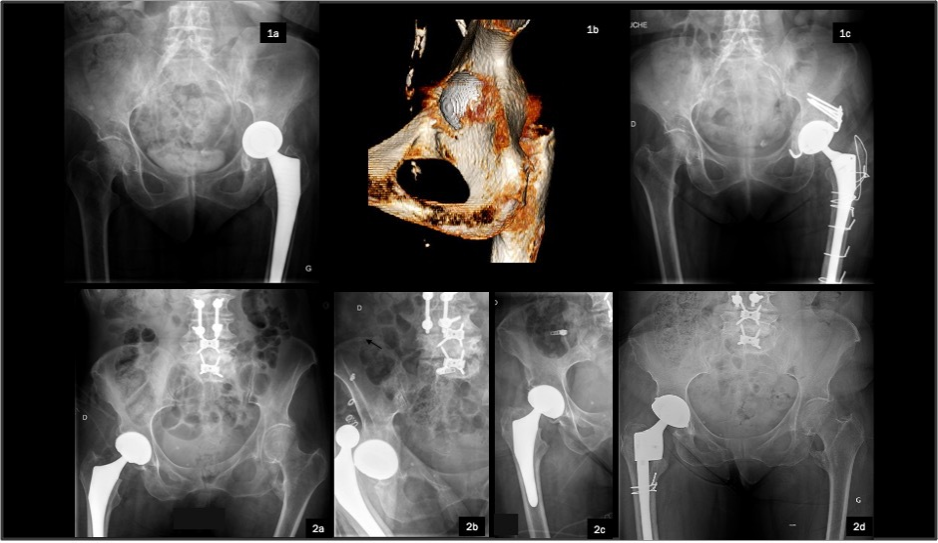
Illustrative cases of re-revision following failed revision total hip arthroplasty (RTHA), shown on anteroposterior pelvic radiographs. Case 1: Re-revision for aseptic loosening. One-stage RTHA in a 67-year-old female for subacute haematogenous methicillin-sensitive *Staphylococcus aureus* infection with acetabular loosening (1a, 1b; shown on a 3D CT scan). Revision with acetabular reconstruction with Kerboull reinforcement ring and bone graft (1c). Septic recurrence at one month managed by modular exchange and intravenous antibiotics. Infection-free at 18 months. Case 2: Recurrent dislocation after uncemented modular dual-mobility THA in a 67-year-old female with prior lumbar fusion and sagittal imbalance (2a, 2b). After failed head exchange and intraprosthetic dislocation (2c) following closed reduction, a full revision was performed using a modular component and a non-modular dual-mobility cup (2d). No recurrence at 18 months.

**Fig. 2:**
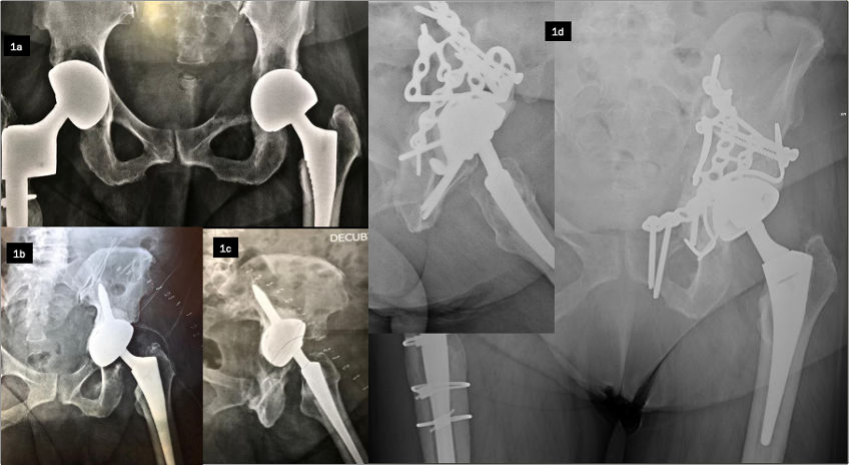
Case 3: Pelvic discontinuity following revision for corrosion in a metal-on-metal bearing (1a) in a 78-year-old female. The initial revision, using a cemented cone-shaped acetabular component and a modular femoral component, was complicated by a fall three weeks postoperatively (1b, 1c), resulting in an anterior column and quadrilateral plate fracture with cup protrusion. A combined surgical approach (posterior and modified Stoppa) enabled implant removal, open reduction and internal fixation with suprapectineal plate, Kerboull reinforcement ring, structural bone graft, and implantation of a cemented dual-mobility cup (1d). Full weightbearing was authorized. There were no complications at 12 months.

**Fig. 3:**
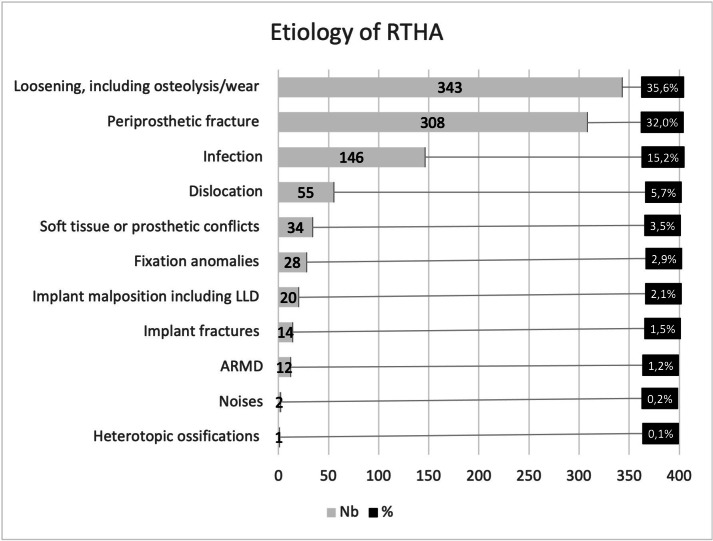
Aetiologies of revision total hip arthroplasty (RTHA). LLD, leg length discrepancy; ARMD, adverse reaction to metal debris.

The most common RTHA aetiology was aseptic loosening, including osteolysis/wear (n = 343, 35.6%), followed by periprosthetic fracture (n = 308, 32%), PJI (n = 146, 15.2%), and dislocation (n = 55, 5.7%) (Figure 1 and Figure 2). Other causes (n = 111, 11.5%) included adverse reactions to metal debris (ARMD), implant fracture, malposition, leg length discrepancy, fixation anomaly, heterotopic ossification, and abnormal noises (Figure 3).

During follow-up, 135 patients (14.0%) experienced failure, defined as re-operation or re-revision. The majority of failures occurred within the first postoperative year (70%).

PJI accounted for 53% of all failures; 61% were classified as ‘new infections’, meaning infections that developed after a revision initially performed for a non-infectious cause. In contrast, patients revised for infection at baseline were also more likely to undergo re-revision for infection compared with other revision indications (p = 0.007).

Instability was responsible for 29% of failures in the subgroup revised for dislocation, with recurrent dislocation occurring in 43.7%. Among these, seven patients required a second re-revision for repeated instability (Table I).

**Table I.** Failure rate and cause according to the initial revision total hip arthroplasty aetiology.

RTHA aetiology	Loosening (n = 343)	PPF (n = 308)	Infection (n = 146)	Dislocation (n = 55)	Other (n = 111)	Total (n = 963)
Failure rate, n (%)	41/343 (12)	31/308 (10.1)	36/146 (24.7)	16/55 (29)	11/111 (9.9)	135/963 (14)
**Failure mode, n (%)**						
Reoperation	1 (2.5)	5 (16.1)	3 (8.3)	4 (25)	2 (18.2)	15 (11.1)
Early re-revision < 1 year	29 (70.7)	22 (71)	29 (80.6)	6 (37.5)	8 72.7)	94 (69.6)
Late re-revision > 1 year	11 (26.8)	4 (12.9)	4 (11.1)	6 (37.5)	1 (9.1)	26 (19.3)
**Failure cause, n (%)**						
Loosening	7 (17.1)	3 (9.7)	1 (2.8)	3 (18.8)	3 (27.2)	17 (12.6)
Periprosthetic fracture	5 (12.2)	4 (12.9)	1 (2.8)	1 (6.2)	1 (9.1)	12 (8.9)
Infection	17 (41.4)	18 (58.1)	28 (77.8)	5 (31.3)	4 (36.4)	72 (53.3)
Dislocation	5 (12.2)	5 (16.1)	5 (13.8)	7 (43.7)	2 (18.2)	24 (17.8)
Other	7 (17.1)	1 (3.2)	1 (2.8)	0	1 (9.1)	10 (7.4)
**Total iterative failure, n**	6	4	8	2	1	
Loosening	1	0	0	0	0	1 (4.8)
Fracture	1	0	0	0	0	1 (4.8)
Infection	1	4	5	1	0	11 (52.4)
Dislocation	3	0	3	1	0	7 (33.2)
Other	0	0	0	0	1	1 (4.8)
Iterative failure rate, n (%)	14.6	12.9	22.2	12.5	9.1	15.6

NS, not significant; PPF, periprosthetic fracture; RTHA, revision total hip arthroplasty.

Fractures represented 30 cases, subdivided into intraoperative fractures (n = 22, including femoral perforations, trochanteric and shaft fractures) and postoperative periprosthetic fractures (n = 8, Vancouver B2 to B3). Six of these led to subsequent failure.

In univariate analysis (Table II), age (p = 0.019), revision rank > R1 (p < 0.001), revision for infection or dislocation (both p < 0.001), use of a constrained cup (retentive design, distinct from dual-mobility) (p < 0.001) or acetabular reinforcement (ring, cages, or augments) (p = 0.011), and the occurrence of early or late complications (p < 0.001) were significantly associated with RTHA failure. No influence was observed for BMI, revision type, surgical approach, extended trochanteric osteotomy (ETO), fixation method, femoral implant, graft use, bearing surface, or intraoperative complications. Mortality was also not associated with RTHA failure.

**Table II.** Univariate analysis of risk factors for revision total hip arthroplasty (RTHA) failure. Comparison between patients who underwent revision of total hip arthroplasty with those without failure.

Variable	RTHA (n = 828)	RTHA failure (n = 135)	p-value*
**Sex, n (%)**			NS
Female	370 (44.7)	62 (45.9)	
Male	458 (55.3)	73 (54.1)	
Mean index PTH year (SD; range)	2007 (10.2; 1973 to 2021)	2009 (9.2; 1984 to 2021)	NS
Mean time to revision, yrs (SD; range)	11.3 (10; 0 to 45)	9.8 (8.8; 0 to 31)	NS
Mean age, yrs (SD; range)	73 (13.5; 14 to 104)	71 (10.6; 37 to 94)	0.019
Mean weight, kg (SD; range)	73.3 (17.9; 35 to 155)	73.7 (17.6; 43 to 127)	NS
Mean BMI, kg/m^2^ (SD; range)	26.2 (5.4; 12.4 to 50)	26.2 (5.3; 17 to 44.3)	NS
**Revision rank, n (%)**			< 0.001
R1	588 (71)	76 (56.3)	
R2	164 (19.8)	32 (23.7)	
R ≥ 3	76 (9.1)	27 (20)	
**Revision type, n (%)**			NS
Bipolar	433 (52.3)	73 (54.1)	
Unipolar femur	139 (16.8)	20 (14.8)	
Unipolar acetabulum	245 (29.6)	37 (27.4)	
Modular components	11 (1.3)	5 (3.7)	
**Atiology of revision, n (%)**			< 0.001
Loosening	302 (36.5)	41 (30.4)	
Fracture	277 (33.4)	31 (23)	
Infection	110 (13.3)	36 (26.7)	
Dislocation	39 (4.7)	16 (11.8)	
Others	100 (12.1)	11 (8.1)	
**Approach, n (%)**			NS
Posterior	818 (98.8)	133 (98.5)	
Anterior	7 (0.8)	2 (1.5)	
Lateral	3 (0.4)	0 (0.0)	
**ETO, n (%)**			NS
Yes	53 (6.4)	8 (5.9)	
No	775 (93.6)	127 (94.1)	
**ETO fixation, n (%)**			NS
Cerclage	31 (58.5)	7 (87.5)	
Plate	22 (41.5)	1 (12.5)	
**Acetabular implant, n (%)**			< 0.001
Standard cup	89 (10.7)	16 (11.9)	
DM cup	735 (88.8)	113 (83.7)	
Constrained cup	4 (0.5)	6 (4.4)	
**Cup fixation, n (%)**			NS
Cement	164 (19.8)	34 (25.2)	
Cementless	664 (80.2)	101 (74.8)	
**Acetabular reinforcement, n (%)**			0.011
Kerboul ring	112 (13.5)	21 (15.6)	
Bursch Schneider ring	25 (3.0)	11 (8.1)	
Augment/custom	14 (1.7)	0 (0.0)	
None	677 (81.8)	103 (76.3)	
**Graft, n (%)**			NS
Allograft	174 (21)	35 (25.9)	
Autograft	77 (9.3)	8 (5.9)	
None	577 (69.7)	92 (68.2)	
**Bearing, n (%)**			NS
Ceramic – polyethylene	57 (6.9)	10 (7.4)	
Ceramic - ceramic	26 (3.1)	3 (2.2)	
Metal polyethylene	745 (90.0)	122 (90.4)	
**Femoral implant, n (%)**			NS
Standard component	364 (44.0)	52 (38.5)	
Long monobloc component	187 (22.6)	22 (16.3)	
Long modular metaphyseal component	192 (23.2)	42 (31.1)	
Long modular neck component	85 (10.2)	19 (14.1)	
**Component fixation, n (%)**			NS
Cement	71 (8.6)	12 (8.9)	
Cementless	757 (91.4)	123 (91.1)	
**Femoral component locking, n (%)**			NS
Yes	179 (21.6)	30 (22.2)	
No	649 (78.4)	105 (7.8)	
**Intraoperative complication, n (%)**			NS
False path	7 (0.9)	1 (0.7)	
Fracture	25 (3.0)	5 (3.7)	
None	796 (96.1%)	129 (95.6%)	
**Early complication < 1 year, n (%)**			< 0.001
Loosening	3 (0.4)	10 (7.4)	
Fracture	5 (0.6)	9 (6.7)	
Infection	25 (3.0)	68 (50.4)	
Dislocation	10 (1.2)	23 (17.0)	
Other	13 (1.6)	8 (5.9)	
None	772 (93.2)	17 (12.6)	
**Late complication > 1 year, n (%)**			< 0.001
Loosening	2 (0.2)	8 (5.9)	
Fracture	0 (0.0)	4 (3.0)	
Infection	6 (0.7)	17 (12.6)	
Dislocation	0 (0.0)	9 (6.7)	
Others	7 (0.9)	3 (2.2)	
None	813 (98.2)	94 (69.6)	
**Death, n (%)**			NS
< 1 year	27 (3.3)	2 (1.5)	
1 to 3 years	10 (1.2)	4 (3.0)	
> 3 years	3 (0.4)	0 (0.0)	
No	788 (95.1)	129 (95.5)	

Bipolar revision = both acetabular and femoral component changed; unipolar femur = femoral component changed; unipolar acetabulum = acetabular component change; modular components = modular exchange of head and/or liner without shell/component change.

* Categorical variables were compared using chi-squared test or Fisher’s exact test. Continuous variables were compared using Student’s *t*-test, Welch’s *t*-test, or Mann-Whitney U test, as appropriate.

DM, dual mobility; ETO, extensive trochanteric osteotomy; NS, not significant.

**Fig. 4:**
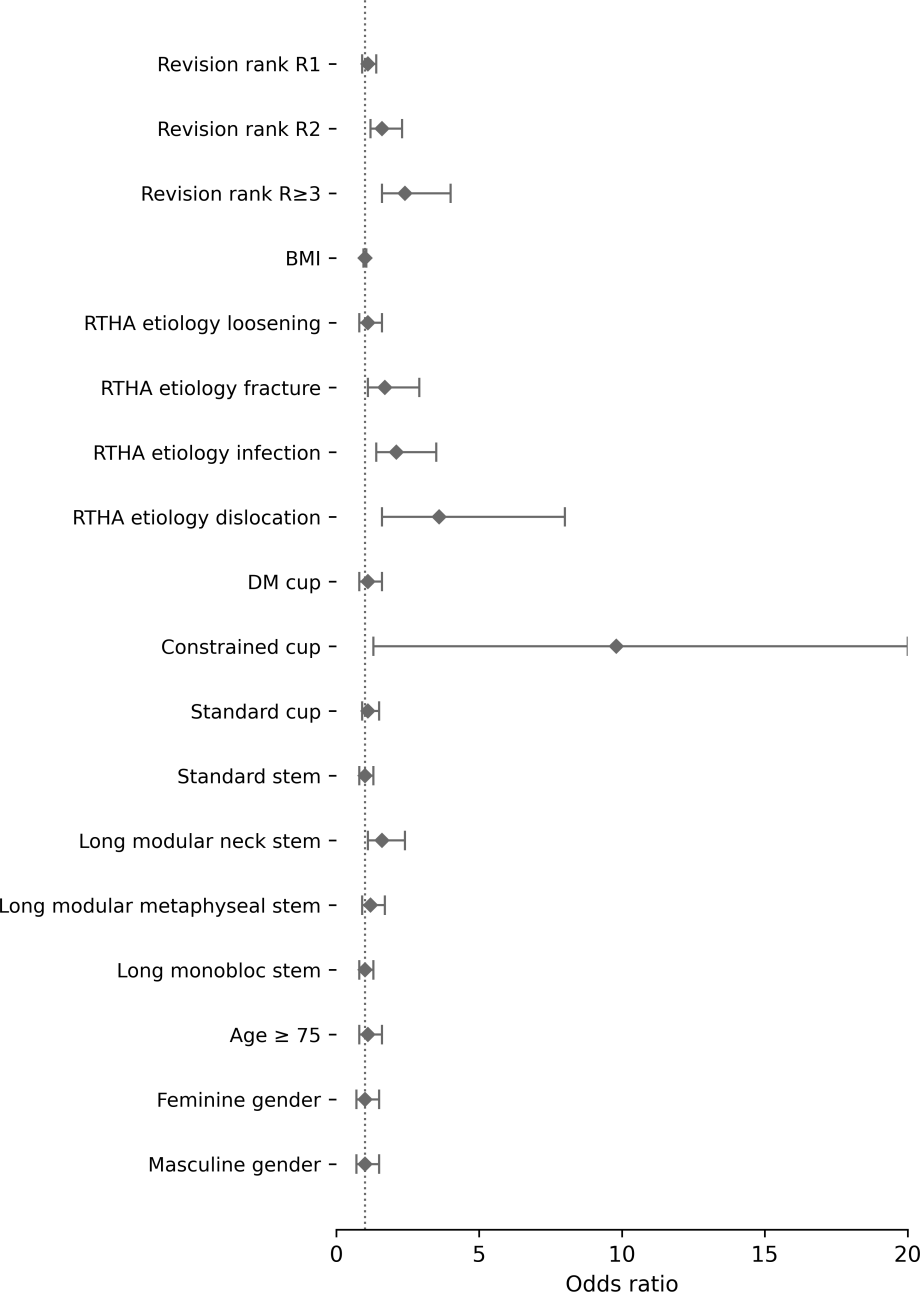
Forest plot showing risk factors influencing failure of revision total hip arthroplasty (RTHA), as determined by multivariate analysis. DM, dual mobility.

In multivariate analysis (Figure 4), age ≥ 75 years (odds ratio (OR) 0.61, 95% CI 0.39 to 0.96; p = 0.033), revision rank ≥ R3 (OR 1.96, 95% CI 1.08 to 3.56; p = 0.026), revision for infection (OR 2.0, 95% CI 1.17 to 3.41; p = 0.011) or dislocation (OR 2.86, 95% CI 1.41 to 5.77; p = 0.004), long modular revision component (OR 2.38, 95% CI 1.17 to 4.85; p = 0.017), and constrained cups (OR 5.38, 95% CI 1.38 to 21.01; p = 0.016) remained independent risk factors for failure.

## Discussion

This multicentre study of 963 patients undergoing RTHA provides contemporary insights into the causes and risk factors for failure after revision surgery. We observed an overall failure rate of 14%, with most failures occurring within the first postoperative year. This figure is consistent with contemporary series: Badarudeen et al^15^ reported a 15.8% iterative failure rate in a large Medicare cohort, while Jafari et al^16^ and Smith et al^17^ described re-revision rates between 12% and 20% in multicentre and registry studies, with infection and instability as the leading causes. Our findings therefore lie within the expected range, while also underlining that the majority of failures occur early, as 70% arose during the first postoperative year. PJI was the leading cause of failure, while recurrent instability remained a major challenge despite the widespread use of dual-mobility (DM) constructs. These findings emphasize the complexity of RTHA and the need for tailored strategies for high-risk patients.

Our results confirm that PJI is now the most common cause of failure following RTHA, accounting for 53% of all failures, including 61% that were new infections after revision for a non-infectious indication. International comparisons show that infection has become a leading indication for revision: 22.7% in Australia, 21.5%, in Sweden, 10.5% in Italy,^18,19^ 15.2% in France, and 11.3% in the USA.^20^

Similar to Jafari et al^16^ and Smith et al,^17^ who also identified PJI as the main cause of iterative failure, our findings highlight its growing impact. Recent registry data indicate that infection-related revisions have doubled in the past 15 years,^21^ particularly during the early postoperative period, as confirmed by Nordic and English series.^22^ Schwartz et al^12^ further reported a 65% increase in revisions for PJI over the last decade.

Beyond incidence, a better understanding of pathophysiology has contributed to the apparent rise in PJI. Studies by Brochin et al^23^ and Kurtz et al^24^ have shown increasing detection rates, likely due to improved microbiological methods and systematic searches for low-virulence organisms such as *Staphylococcus epidermidis* and *Cutibacterium acnes*,^25^ which were previously underestimated.^21^ Renard et al^26^ reported occult infection in 7% of presumed aseptic revisions, underscoring the importance of systematic screening. Together, these findings emphasize that infection remains the foremost obstacle to long-term success in revision surgery and requires ongoing optimization of both prevention and management strategies.

Instability was the indication for revision in only 5.7% of our cohort, reflecting the widespread French adoption of DM cups, compared with more than 20% in the USA prior to Food and Drug Administration (FDA) approval of DM in 2009.^27,28^ In our series, the protective effect of DM constructs on RTHA survivorship was confirmed, consistent with international literature showing that DM significantly reduces dislocation risk without increasing loosening^29^ or infection rates.^30^

Nevertheless, dislocation remains a critical issue in selected cases. Revision for instability was associated with a 29% failure rate in our cohort, with recurrent dislocation occurring in 43.7% of cases, particularly in patients with chronic instability, multiple previous operations, or abductor deficiency. These results are consistent with published iterative dislocation rates ranging from 21% to 39%.^31^ Intraprosthetic dislocation, although rare, was also observed in two cases (0.2%), both involving modular DM cups, in line with reported rates around 1%.^32^

When recurrent instability persists, constrained acetabular components are often considered. However, their use was associated with a 60% failure rate in our series, mainly due to loosening and infection. This reflects both the inherent complexity of these cases and the known limitations of constrained designs, as previously reported by Della Valle et al,^33^ Hernandez et al,^34^ and others.^33^ While constrained liners may prevent dislocation in the short term, they should be reserved for highly selected patients given their poor long-term durability.

Finally, periprosthetic fracture, often under-reported in international series, emerged as the second most common indication for revision in our cohort (32%). This proportion is higher than in Australia (21.8%) or Sweden (9.2%), and represents a striking increase compared with a previous French multicentre study reporting 11.8% in 2010.^35^ This trend likely reflects demographic changes, longer life expectancy, and the growing prevalence of hip arthroplasty, and underlines the need to anticipate an increasing burden of fracture-related revisions in the future.

The strengths of this work include its large sample size, multicentre design, and comprehensive dataset, which captures both reoperations and re-revisions, events often missed by national registries. Our study also distinguishes between early and late failures and identifies new compared with recurrent infections, offering clinically relevant granularity.

Limitations include its retrospective nature and reliance on coding systems, with potential classification bias and incomplete data for comorbidities, functional outcomes, and mortality beyond one year. Another limitation relates to implant design data. Although we documented the use of dual-mobility constructs and identified intraprosthetic dislocations in two modular cups (MDM X3; Stryker, USA), detailed information on the breakdown between monobloc and modular dual-mobility implants, as well as manufacturer-specific data, was not consistently available across all centres. As a result, our analysis could not explore potential differences in outcomes according to implant subtype. This limitation should be considered when interpreting our results.

Importantly, failures after revision are time-dependent events, but due to incomplete availability of index THA dates across centres, Kaplan-Meier or Cox regression analyses could not be performed. We therefore relied on logistic regression, and our results should be interpreted within this context. Nonetheless, the finding that 70% of failures occurred within the first year provides valuable information about the critical early postoperative period.

In conclusion, despite advances in implant technology and surgical techniques, revision THA remains associated with substantial risk of iterative failure. PJI has emerged as the predominant challenge, while recurrent instability continues to pose difficulties in selected patients. The widespread use of DM cups in France has reduced the burden of dislocation, but constrained liners and modular components remain associated with high failure rates. The rising incidence of periprosthetic fractures adds further complexity.

These findings underline the need for meticulous preoperative planning, rigorous infection prevention, and tailored implant selection, particularly in high-risk patients. Future prospective studies incorporating survival analyses and functional outcomes will be essential to refine predictive models and improve long-term durability after revision arthroplasty.

**Take home message**

- This study identifies key patient-, pathology-, and implant-related factors associated with failure after revision total hip arthroplasty, highlighting the predominant roles of periprosthetic joint infection and recurrent instability.

- Recognition of these high-risk profiles may help surgeons refine preoperative planning, implant selection, and perioperative strategies to reduce early re-failure.

- In particular, the findings underscore the need for meticulous management of infection and instability in complex revision cases.

## Data Availability

The datasets generated and analyzed in the current study are not publicly available due to data protection regulations. Access to data is limited to the researchers who have obtained permission for data processing. Further inquiries can be made to the corresponding author.
